# Color-selective holographic retroreflector array for sensing applications

**DOI:** 10.1038/lsa.2016.214

**Published:** 2017-02-24

**Authors:** Rajib Ahmed, Ali K Yetisen, Seok Hyun Yun, Haider Butt

**Affiliations:** 1 Microengineering and Nanotechnology Laboratory, School of Engineering, University of Birmingham, Birmingham B15 2TT, UK; 2 Harvard Medical School and Wellman Center for Photomedicine, Massachusetts General Hospital, Cambridge, MA 02139, USA; 3 Harvard-MIT Division of Health Sciences and Technology, Massachusetts Institute of Technology, Cambridge, MA 02139, USA

**Keywords:** Bragg gratings, corner cube retroreflectors, diffraction optics, holography, sensors

## Abstract

Corner cube retroreflectors (CCRs) have applications in sensors, image processing, free space communication and wireless networks. The ability to construct low-loss wavelength filters embedded in CCRs can enable the development of wavelength multiplexing, tunable lasers and photonic integrated circuits. Here we created an ~10-μm-thick holographic corner cube retroreflector (HCCR) array that acted as a color-selective wavelength filter and diffracted light at broad angles. Angle-resolved spectral measurements showed that the Bragg peak of the diffracted light from the HCCR array could be tuned from 460 to 545 nm by varying the incident angle. The HCCR array also exhibited a wavelength-selective tuning capability based on the rotation angle in the visible spectrum. HCCRs projected holographic images with the rotational property in the far field. The utility of the HCCR was demonstrated as optical temperature and relative humidity sensors that produced a visible colorimetric response for rapid diagnostics.

## Introduction

Corner cube retroreflectors (CCRs) consist of three mutually perpendicular intersecting flat surfaces that directly reflect incident light back to its source^1, 2^. The incident light is internally reflected three times in CCRs, and their directional feature is independent of the incident angle. CCRs have applications in satellite communication, laser components and antennas^3, 4, 5^. In particular, the phase conjugation property of a CCR array has been widely used for wavefront correction and for enhancing the resolution in image processing^6, 7^. The phase conjugation property of CCR array surfaces can also be utilized in wavefront sensing, phase-conjugated interferometry and Fourier transform holography^8, 9, 10^. Recently, microelectromechanical retroreflectors have been fabricated for miniaturized applications^11, 12^. CCRs fabrications are typically based on micromechanical processing or photolithography^13, 14, 15, 16^. These fabrication approaches are costly, expertise dependent and time-consuming, thus limiting their scalability for practical applications in photonics.

Here we show the development of a three-dimensional HCCR array using Denisyuk reflection recording. The HCCR array does not exhibit all the analogous optical properties when compared with CCRs. However, the HCCR array is planar and thinner, functions as a narrow-band wavelength filter and exhibits the rotational property. In this work, we analyze the directional reflection properties of the CCR array through numerical modeling; their holographic images are recorded using silver halide chemistry. We also present a unifying view regarding the rotational property of HCCRs. Angle-resolved reflection measurements of the HCCRs show a reasonable diffraction efficiency (DE), along with the directional color filtering of broadband light. Finally, we demonstrate the utility of HCCR in colorimetric temperature and relative humidity (RH) sensing. This work is the first report of the production of HCCR arrays, which may have potential applications in compact optical systems and sensors.

## Materials and methods

### Holographic retroreflector fabrication

**Figure 1:**
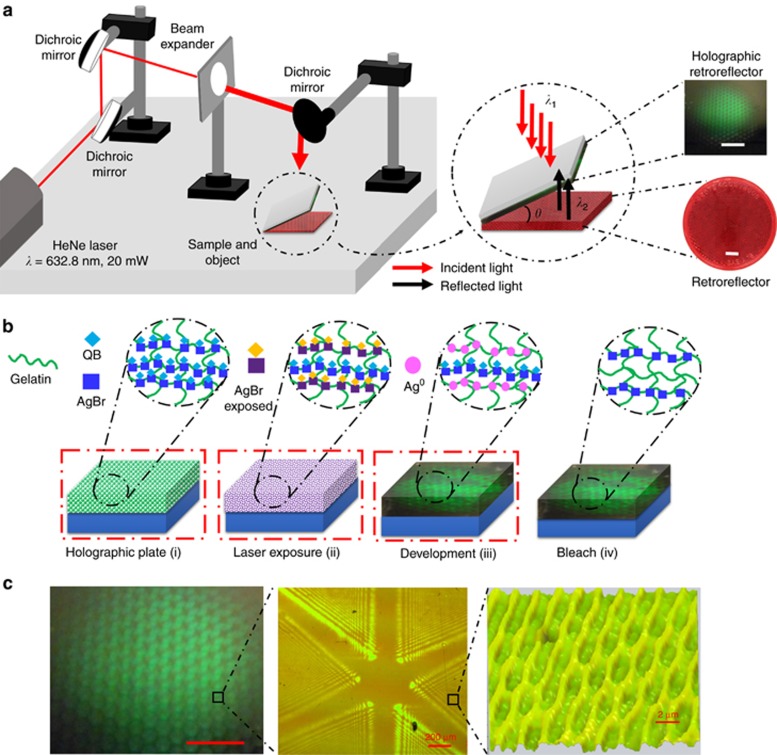
Holographic recording of a HCCR array in light-sensitive media. (**a**) The experimental setup in Denisyuk reflection mode. Scale bars=1 cm. (**b**) Holographic retroreflector array preparation: (i) using a gelatin matrix containing photosensitized AgBr NCs, photosensitization was performed in the presence of quinaldine blue (QB); (ii) gelatin matrix with AgBr NCs was exposed to a laser beam (632.8 nm) to record an image of a retroreflector array; (iii) photographic developer was used to reduce the exposed AgBr NCs to silver metal (Ag^0^) NPs; (iv) the hologram was bleached to increase DE, and Ag^0^NPs were converted back to AgBr NCs. (i–iv) were performed under green safe lighting. (**c**) Top view of the holographic retroreflector and its surface geometry, consisting of triangular sections on the surface and an internal multilayer structure. Scale bar=1 cm.

The HCCR array was fabricated using Denisyuk reflection holography. A HeNe laser beam (*λ*=632.8 nm, 20 mW) was passed through a beam expander (*Ø*=6 cm) and a collimator. In Denisyuk reflection mode, an image of an array of CCRs (object) was recorded in a light-sensitive emulsion (Figure 1a). The holographic plates (2cm × 2 cm) consisted of a gelatin emulsion containing light-sensitive silver bromide (AgBr) nanocrystals (NCs) (*Ø*=10–20 nm) conjugated to quinaldine blue (1,1'-diethyl-2,2'-carbocyanine) dye. The sample was exposed to a laser beam after being tilted (*θ*≤5°) from the mirror surface plane. The tilt angle created a slanted interference pattern in the recording medium, which separated the diffracted light from the specular reflection (off the glass substrate). Low tilt angles reduced the effect of the transmission grating and increased the DE^17^. An index matching fluid was used to reduce the transmission grating effect. The interference pattern formed in the holographic plate involved two beams: incident (reference) beam, *λ*_1_, and reflected (object) beam, *λ*_2_. A corner cube reflector array (the dimension of each CCR is ~0.2 cm) was used as an object to record the interference pattern within the gelatin matrix.

The sample development was based on silver halide chemistry (Figure 1b)^18, 19^. The gelatin contained AgBr NCs that had been optimized for high-resolution holography. Glass was used as a substrate, and its surface was functionalized using (3-aminopropyl)-triethoxysilane in acetone (1:100, v/v). Gelatin is a transparent medium and has flexible pores that can accommodate AgBr NCs and Ag^0^ nanoparticles (NPs). The gelatin holographic plates were pre-swollen by immersing them in a bath of aqueous solution of triethanolamine (10%, v/v) for 1 min and then dried in a cold airflow at 60% RH for 1 h. Upon exposure to laser light, a latent image was formed in the emulsion through the disruption of the AgBr ion-pair with a laser light, that is, AgBr+hγ→Ag^+^+Br^−^+e^−^, and subsequent silver atom formation at an electron trap site in the AgBr NC, that is, Ag^+^+e^−^ → Ag^0^^20^. A photographic developer containing 4-(methylamino)phenol hemisulfate (pH ~12) was used to amplify the latent image in the AgBr NCs into silver metal (Ag^0^) NPs to form a multilayer structure of the visual image. Bright fringes (antinodes) in the standing wave were amplified as Ag^0^ NPs. The emulsion was bleached (KBr and CuSO_4_) by converting the Ag^0^ NPs back to transparent AgBr NCs. Bleaching increases the DE and reduces light scattering^18, 21^. After drying, the HCCR exhibited efficient green diffraction, and the surface contained micropatterned arrays (Figure 1c). The diffraction from the HCCR effect was due to the periodic arrangement of AgBr NCs within the gelatin matrix, obeying Bragg’s law, and the surface structures contributed to angular anisotropy.

### Computational modeling, phase conjugation and rotational property of CCR array

To understand the optical interference patterns, which produce the HCCRs, optical simulations of an array of CCRs were performed (Supplementary Information, Supplementary Fig. S2). The reflection and directional properties of the CCRs arrays were analyzed using the finite element method. The structure of the retroreflector was considered as a two-dimensional triangular grating (Supplementary Information, Supplementary Fig. S2). To obtain approximation results, the triangular grating’s spacing and depth were considered to be ~15–20 times larger than the incident wavelength, *λ* (*d*>>*λ*). For simplicity, a two-dimensional simulation was performed using triangular meshes (the maximum mesh size was approximately one-tenth of the input wavelength) with a fine domain structure, for a total of 23 698 meshing elements^22, 23^.

The phase conjugation and rotation property of a CCRs/HCCR array are based on wavefront analyses of incident and reflected/diffracted light. Consider an arbitrary incident wave under paraxial approximation 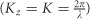:


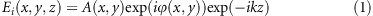


The reflected light from the CCRs array is


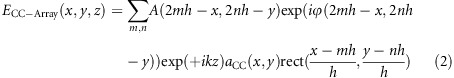


where rect(*x*, *y*) is a rectangular function equal to 1, where abs(*x*, *y*)<1/2, and is 0 otherwise; and *a*_cc_(*x*,*y*) is a scalar quantity known as the aperture function and indicates the reflected beam amplitude (Supplementary Information ‘Directional, Phase Conjugation and Rotational Property of CCR Array’). The wavefront reflected from the CCR array surface showed a reversed phase but the same amplitude sign as that of the incident wavefront. This might have been due to the triangular surface of the CCRs array (side view), where the reflected wavefront was disrupted, cut into segments and shifted along the direction of propagation, showing analogous properties to those of diffraction gratings and Fresnel lenses^6^. The rotation property of CCRs, based on wavefront analyses of incident and reflected light, was also studied (Supplementary Information ‘Directional, Phase Conjugation and Rotational Property of CCR Array’). If the incident holographic image rotates in the clockwise direction, the far-field projected image from CCR and HCCR rotates in the reverse direction (Supplementary Information, Supplementary Fig. S2 and Supplementary Movie 1). The rotational property of the HCCR array was based on light diffraction from the HCCR surface. If the sample rotated (for a broadband light source) in the clockwise direction, the diffracted color light from the HCCR array rotated in the anticlockwise direction (Supplementary Information, Supplementary Fig. S2 and Supplementary Movie 2). The rotational property was found to be valid for the CCR and HCCR retroreflector.

## Results and discussion

### Optical characterization

**Figure 2:**
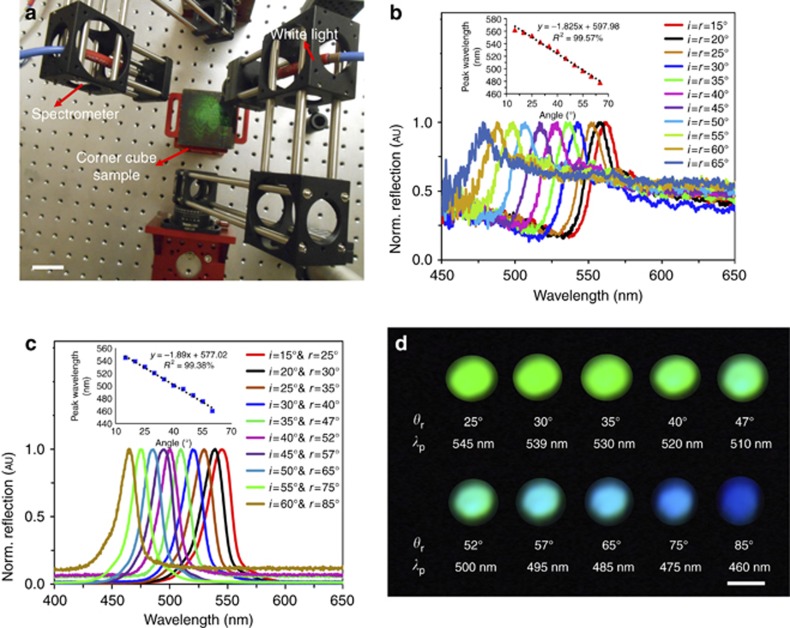
Angle-resolved measurements of the HCCR array. (**a**) Goniometer setup used to measure reflected light from the retroreflector under white light illumination. Scale bar=6 cm. (**b**) Normalized reflected light as a function of incident angles (*i*=15°–65°); inset shows linear fitting of reflected peak wavelength as a function of incident angles. (**c**) Normalized monochromatic diffracted light as a function of incident angles (*i*=15°–60°); inset shows linear fitting of diffracted peak wavelength as a function of incident angle. (**d**) Diffracted colors at different receiver positions *θ*_r_ (=*r*) and peak wavelengths (*λ*_P_). Scale bar=2 cm.

The HCCR arrays were spectroscopically characterized in reflection mode. The sample was illuminated using a broadband light source (450–1100 nm). An Ocean Optics 2000 spectrometer (450–1100 nm, 0.2 nm resolution) was used to measure the reflected and diffracted light. A goniometer setup was utilized to obtain spectra at different incident angles to capture the maximum reflection and diffraction peaks (Figure 2a). The reflection readout of the holographic retroreflector was found to be highly dependent on the incident angles (Figure 2b). In response to incident light, an intense broadband reflection signal (specular reflection from the substrate) was detected, as was narrow-band diffraction. The properties of the diffracted light were dependent on the incident angles and sample position.

The reflected broadband signal also displayed maximum peaks (Figure 2b). The peak blueshifted linearly as the incident angle was increased from the normal. This peak might represent the resonance of Ag^0^ NPs within the gelatin. In addition, the narrow-band diffracted light was analyzed by varying the incident angles and moving the spectrophotometer probe to measure the maximum intensity (Figure 2c). The holographic retroreflector acted as a sensitive color filter by separating colors from the incident white light, depending on the incident angles. For the reflected white light (zero order), the incident and reflection angles were equal. However, for the diffracted narrow-band light (higher orders), the incident angles were different from the collection/diffraction angles, and the diffraction angles increased nonlinearly for larger incident angles. This was due to the volumetric multilayer structure in the HCCR array^17^. The separation between the reflected and diffracted light was ~10°. However, this separation increased with larger incident angles from 12° to 25°.

The reflected spectra mainly showed broadband light, with peaks in the range of 506–562 nm (green) and 478–497 nm (blue) for incident angles between 15° and 65°. The peak that was originally at 562 nm (*i*=15°) shifted linearly from 558 to 478 nm for *i*=20°–65°, in 5° increments, respectively. For larger incident angles, lower-intensity peaks were measured, which could have been due to the decreasing diffraction intensity. The HCCR array showed 20–40 nm lower Bragg peaks because the lattice spacing of the multilayer grating contracts due to bleaching and exposure in a swollen state. Figure 2c shows the normalized diffracted light spectra as the incident angle was changed. The peak blueshifted, and its intensity decreased at broad incident angles. Diffraction also showed a linear trend at different incident angles. The diffracted light’s peak wavelengths (*λ*_p_) were measured at different receiver positions, *θ*_r_ (=*r*) (Figure 2d). The diffracted light shifted from green to blue and then to violet as the incident angle was increased. The shift to shorter wavelengths was due to the venetian blind effect^24^. At broad incident angles, the lattice spacing seen by the white light may have been smaller compared with that at lower incident angles. The intensity of the diffracted light was low for angles >65°.

**Figure 3:**
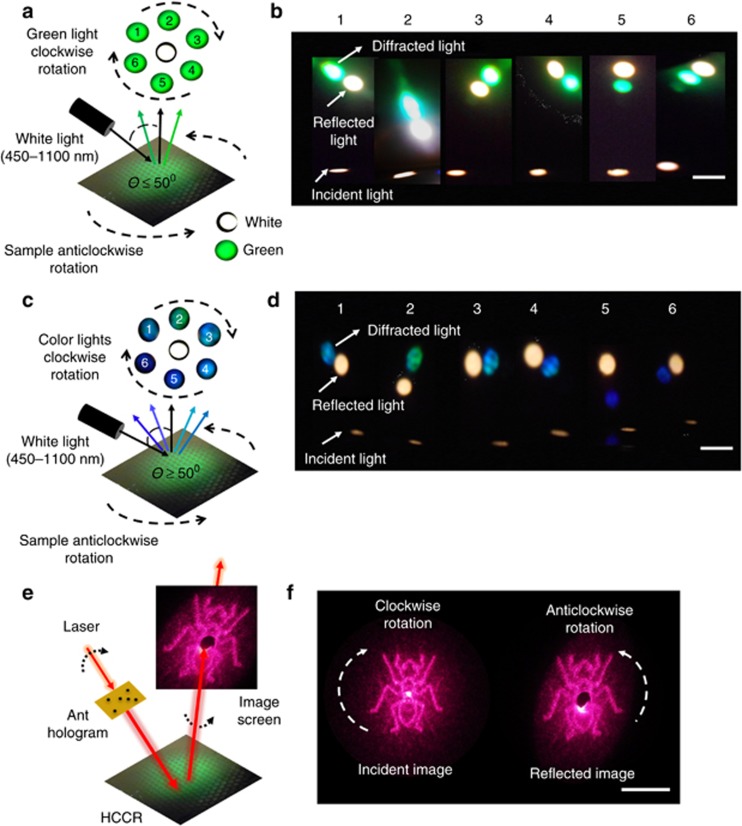
Rotational and color filtering characteristics of the HCCR array. (**a**) Incident light, specular reflection and diffracted green light. (**b**) With *i*≤50°, clockwise rotation of the sample, green diffraction spot rotated anticlockwise. Scale bar=2 cm. (**c**) Incident light, specular reflection and diffracted narrow-band light (various colors). (**d**) Narrow-band diffraction variation, with sample rotation at incident angles *i*≥50°. Scale bar=2 cm. Supplementary Information (Supplementary Movie 2) shows the variation of the Bragg diffraction as the sample is rotated. (**e**) Experimental setup to analyze rotational property of the HCCR array. (**f**) Holographic incident image (left) from a laser beam and resultant projected image on a screen (right). Scale bar=2 cm.

Spectral measurements were performed using fixed incident angles (*i*≤50° or ≥50°) and rotated positions. The Bragg peaks of the diffracted light changed as the sample was rotated. With anticlockwise rotation of the HCCR array, diffracted light rotated in the clockwise direction (Figure 3a and 3b). This occurred for the diffracted green light; the reflected white light remained in the same position (*i*≤50°). In retroreflectors, white light also rotates in this position. Similar results were also found with higher incident angle (*i*≥50°), the clockwise rotation of the HCCR array, where diffracted light rotated in the anticlockwise direction (Figure 3c and 3d). This rotation property was due to the tilt of the multilayer grating recorded within the gelatin matrix. The corner cube multilayer structure splits the wavefront and reverses the phase at every point in the reflected wave (Equation 2). As the HCCR array was rotated, the grating diffracted light at different collection angles. The HCCR array filters and diffracts the green color for incident angles less than 50°. However, it also diffracted different color combinations (green, blue, violet and their mix colors) for incidence angles >50° (Figure 3d). This is of interest as the HCCR array can be used as a tunable optical filter to select colors depending on the incident and azimuthal angles.

Figure 3e shows the experimental setup used to characterize the rotational property of the HCCR array. A red laser beam (632 nm, 20 mW) was holographically shaped to form an image of an ant and was irradiated onto the HCCR array. A slight incident angle was chosen to record the reflected image separately. The reflected holographic image was projected onto a white screen with a centered perforation (*Ø*=10 mm). The reflected zero-order beam passed through the opening, and the holographic phase conjugated image (ant) was captured on the screen. Figure 3f shows the incident holographic image (left side) from a laser beam and reflected image (right side). The holographic projection also obeyed the rotational property: upon the clockwise rotation of the incident image, the reflected image also rotated anticlockwise (Supplementary Information, Supplementary Movie 1). The rotational property of the ant-hologram was also found to be valid with a single CCR/HCCR and to work properly with other visible light sources (for example, blue and green).

**Figure 4:**
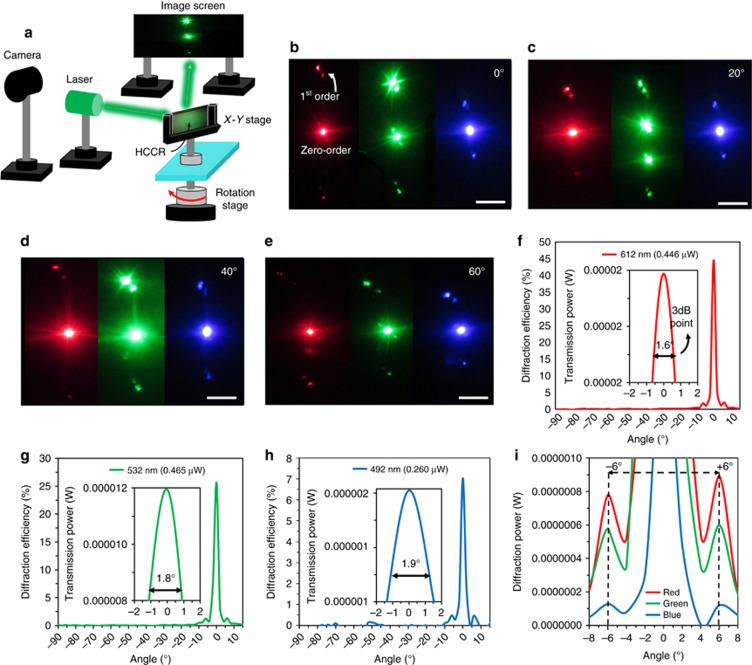
Characterization of visible diffraction from the HCCR array. (**a**) Experimental setup used for shining laser light on the HCCR array at various angles and projecting the diffracted light on a screen. (**b**–**e**) The diffraction patterns in response to red, green and blue laser light, irradiated at incidence angles from 0° to 60°. Scale bars=2 cm. (**f**–**i**) Angular DE measurement of the HCCR. The DE and intensity in response to red, green and blue laser light at rotation angles from −90° to +90°. The DE and intensity decrease as wavelength increases; however, the angular spread of the non-diffracted spot at the 3 dB point increases.

As the HCCR array diffracted various colors at different positions, an angle-dependent Fraunhofer diffraction analysis was performed^25, 26^. The far-field diffraction patterns produced by the HCCR array in response to red, green and blue laser beams were studied. Figure 4a shows the experimental setup: the HCCR array was placed vertically on top of a rotation stage, equidistant from the laser source and the screen (~40 cm). The incident angle of the laser beam was varied from 0° to 60° by rotating the HCCR array. The diffracted light was projected onto a semi-transparent screen. Figure 4b–4e shows far-field diffraction patterns. The distance between the first-order and zero-order decreased as the incident beam shifted to shorter wavelengths from red to blue, thus obeying Bragg’s law.

At low incident angles, a highly intense green diffraction pattern was produced compared with the red and blue light. This was due to the overlap between the Bragg resonance of the HCCR array and the incident green laser beam. However, as the incident angle was increased, the blue diffraction pattern increased in intensity, which agreed with the results in Figure 2c and 2d, showing a blueshift with an increasing incidence angle. This capability showed that the HCCR arrays worked on the basis of diffraction, producing various orders compared to the conventional reflective CCRs and displaying an angular wavelength-selective image projection.

Angular dependent diffraction measurements of the HCCR were studied in response to red, green and blue laser light at rotational angles from −90° to +90° (Figure 4f–4i). The sample holder and light source were supported by a stepper motor, which was rotated horizontally by 360° (1° step size). An optical power meter was placed normally to the rotational stage to measure the diffraction power. Figure 4f–4i shows the DE and power as a function of the rotation angles. Maximum power and efficiency was measured using a red laser at the non-diffracted zero order. Symmetrical first-order diffraction peaks were observed on each side of the zero-order. The DE was measured as (*P*_diff_/*P*_in_) × 100%, that is, the ratio of the diffracted power (*P*_diff_) and incident power (*P*_in_)^27, 28, 29^. The average diffraction power and efficiency of the 1st-order peaks contributed approximately 8% and 4%, respectively, to the overall diffraction power and efficiency for the red laser compared with the non-diffracted zero-order. Similarly, the average diffraction power and efficiency of the first-order green and blue peaks contributed 5%, 4% and 6.6%, 7%, respectively. Moreover, the average diffraction power of the first-order peaks reduced to 50% and 80% for the green and blue laser, respectively, compared with the first-order red diffraction (Figure 4i). The DE and the diffracted power were reduced with shorter incident beam wavelengths. Similarly, the angular spread of the non-diffracted spot at the 3 dB point decreased as the wavelength increased (inset in Figure 4f–4h). This might have occurred because the spectral range of the gelatin matrix containing the AgBr emulsion was 580–650 nm and the sample was recorded using a red laser^30^. Power fluctuations were also measured between the symmetrical peaks (Figure 4i). Distortion at lower order diffracted peaks was also found in the literature^31^. The HCCR showed a lower diffraction efficiency compared with the normal CCR array, but it presented the added advantage of being flat and compact (~10 μm). The optical property of the HCCR is similar to that of the other diffractive optical elements (for example, Bragg grating) and shows less intense diffraction^6, 29^. The first-order diffracted red, green and blue light showed almost the same distance (6°) from the zero-order. The difference was visible in the far field (Figure 4b–4e). The resolution of the HCCR was measured as 5052 lp mm^−1^, with a maximum NC grain size of ~20 nm. The resolution of the recorded hologram was calculated as *γ*=(2*n*sin (*δ*/2))/*λ*, where *γ* is the spatial frequency, *n* is the refractive index of the emulation (1.6), *λ* is the recording wavelength (632.8 nm) and *δ* is the angle between the object and reference beam (175°). In the present work, visible light diffraction and broadband light were chosen because they were readily available and suitable for visual detection by the eye. The diffraction property can be extended to the ultraviolet (UV) and near-infrared (IR) region of the spectrum. For this, the hologram needs to be recorded at shorter or longer wavelengths using UV or IR wavelengths, where the diffraction peak of the multilayer gratings is limited by Bragg’s law. In addition, to tune the Bragg peak, pseudo-color holograms can be produced by pre-swelling or shrinking the recording media.

### Temperature and RH sensing

**Figure 5:**
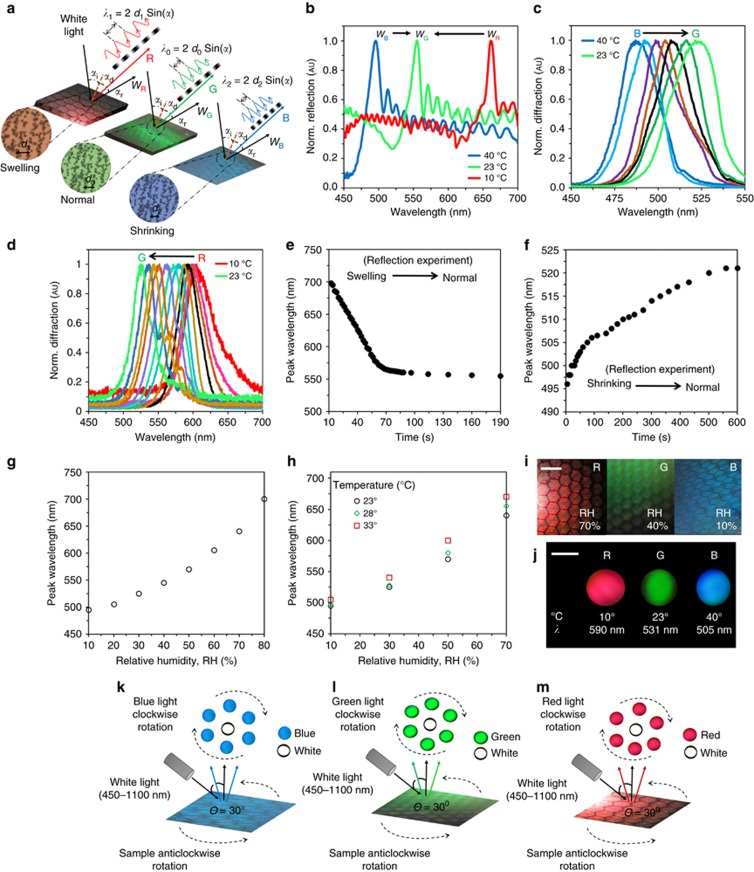
Colorimetric HCCR temperature and RH sensors. (**a**) Conceptual diagram of the HCCR temperature and RH sensor. The temperature and RH variation act as external stimuli for the sensor, swelling or shrinking the multilayer lattice spacing of the holographic matrix. (**b**–**d**) The normalized reflection and diffraction intensity as a function of variation in temperature. White light illumination with incident angle was from 0° to 30° during the diffraction experiment. (**e**, **f**) Reflected peak wavelength shift as a function of time during swelling/shrinking to initial conditions, respectively (Supplementary Information,Supplementary Movie 3). (**g**, **h**) Reflected peak wavelength as a function of RH variation. (**i**) Holographic color variation with RH variation. Scale bar=0.4 cm. (**j**) Far-field diffracted color with temperature variation (Supplementary Information,Supplementary Movie 4). Image projection screen was 30 cm away from the sensor. Scale bar=2 cm. (**k**–**m**) Rotation property as a function of RH/temperature variation.

To demonstrate the utility of the HCCR, we show an application in sensing. Figure 5a shows the conceptual diagram of the HCCR temperature and RH sensors. The Bragg peak of the diffracted color light (*α*_d_) and reflected white light (*α*_r_) from AgBr NC multilayers of the holographic polymer film responds to variation in temperature and RH. The sensing principle is based on AgBr NC multilayer structures that can swell or shrink in response to external stimuli. The grating structure acts as an optical transducer and can quantify the external stimuli by shifting the Bragg peak in the visible spectrum. The HCCR sensor responds to the incident white light and diffracted green (G) light at the steady-state condition (no stimuli); it acts as a reference between transition conditions such as swelling or shrinking period due to external stimuli. At high temperature and lower RH values, the lattice spacing of the internal multilayer shrinks, which shifts the Bragg peak to shorter wavelengths.

The AgBr multilayer lattice spacing became smaller for increasing temperature/lower RH, and the response curve shifted to shorter wavelengths (Blue, B), obeying Bragg’s law (*λ*=2*d*sin*α*; *α*=incident angle from sample normal, *d*=lattice spacing and *λ*=diffraction wavelength). Figure 5b–5d shows the reversible HCCR sensor response as a function of temperature variation, which also holds for RH variation. The HCCR tended to retain moisture at low temperatures (10 °C), and the normalized peak wavelength shifted from red to green while shrinking to the normal condition. Similarly, HCCR shrunk at higher temperatures (40 °C) and shifted from blue to green while shrinking to the normal condition. Figure 5e and 5f show Bragg peak wavelength shifts as a function of time. Larger Bragg peak shifts were found initially due to higher temperature difference between the holographic polymer and environment. The sensitivity of the HCCR sensor was measured using the gradient of the Bragg peak shift response curve (Supplementary Information ‘Temperature and Relative Humidity Sensitivity Measurement’). The sensitivity of the sensor was measured as ~4 nm °C^−1^ (23 °C) and 1 nm (40% RH).

Figure 5g and 5h illustrates the HCCR sensor response as a function of RH variation. The peak wavelength shifted toward longer wavelengths as the RH increased. This was due to the swelling of the recorded HCCR medium and the consequent increase in fringe spacing (Figure 5g). In a real scenario, sensing complexity will be increased when both the temperature and RH change simultaneously. The peak wavelength shift-dependent function of temperature and RH variation is


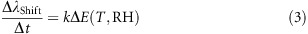


where *k* is the proportionality constant, which is related to the heat transfer coefficient between the recording medium (HCCR matrix) and the environment, and Δ*E*(*T*, RH) is the change in the environment as a function of temperature and RH. The sensor showed a longer wavelength shift with larger RH variation. To evaluate temperature dependence, the peak wavelength shift was measured with RH variation in a controlled environment chamber (Supplementary Information ‘Temperature and Relative Humidity Sensitivity Measurement’). The response curve showed (Figure 5h) a small temperature dependence at lower RH values. A larger wavelength shift occurred due to the increasing temperature and RH variation. Hence, at higher temperatures, the HCCR matrix expanded by a greater extent for the same RH change. The graphs of wavelength shift vs RH are temperature-dependent; this can be used to sense both temperature and humidity changes. Therefore, the HCCR showed RGB visible color selectivity in response to the temperature or RH variation that was visible to the naked eye (Figure 5i). The far-field sensor readout projection also showed diffracted color change with a variation in temperature (Figure 5j). Variation in the external stimuli finely tuned the lattice spacing, which resulted in the Bragg peak in the visible spectrum. The rotational property of HCCR was also tested during the sensing experiments. The rotational property of the HCCR based on the diffraction property was also valid with RH and temperature variation. The diffracted red, green and blue light (based on RH and temperature) rotated clockwise, with sample rotation in the anti-clockwise direction (Figure 5k–5m). This property of the HCCR sensor is unique, thus offering potential applications in colorimetric sensing and sample tilt/rotation monitoring.

## Discussion

In a conventional CCR array, the directional property is imperative, where an incident light experiences three mirror reflections and returns toward the light source^6^. However, the HCCR array does not produce the three-time reflection effect and return light toward the light source; the light is instead diffracted, as occurs for diffraction gratings and Fresnel lenses, which obey Bragg’s law^6^. Hence, unlike the conventional CCRs, one cannot see the reflection of their own eye in the fabricated HCCR plate. However, the rotation property of HCCR based on the diffraction property was demonstrated to characterize the HCCR array. An array of HCCRs was also fabricated without a tilt angle during interference field exposure (Supplementary Information,Supplementary Fig. S5). The holographic CCR array with a tilt angle of 5° from the surface plane and CCR array with no tilt angle exhibited similar optical effects and spectral readouts.

Practically, distinct properties of the HCCR were found for applications in photonic devices. First, the HCCR array showed wavelength tunability in its diffraction at different incident angles. Hence, it can be used as a visible color filter, wavelength-selective diffraction grating, or printable optical device^31, 32^. In addition, the rotational property displayed by the HCCR offers opportunities as a tunable and color-selective mirror in adaptive micro-optics, endoscopic tweezers and wavefront sensing^8, 33, 34^. The HCCR array can also be used as a security hologram, with its phase conjugation/rotational property being difficult to replicate and easy to verify^35, 36, 37, 38^. Such holograms can also be used as wide field of view optical biosensors by fabricating them in functionalized hydrogels, which expand and contract due to changes in analyte concentrations^17, 19^.

## Conclusions

The HCCR array (10 μm thick) has been developed using a simple top-down fabrication approach. This is the first report of planar CCRs and HCCR arrays produced with silver halide chemistry in Denisyuk reflection mode, which can be used for the highly efficient diffraction/filtering of particular colors depending on the incident angle. The HCCR array is operated based on Bragg diffraction and exhibited reflection properties and additional wavelength-selective filtering compared with CCRs. The HCCRs exhibited wavefront conjugation, along with tunable wavelength-selective filtering, and acted as a colorimetric temperature and RH sensor whose readouts were visible to the naked eye. The rotation property is proposed and showed experimentally to characterize the HCCR array. The diffraction intensity of the HCCR array varied with incident angle and wavelength. The presented fabrication approach is simple and allows the production of customized HCCR arrays for different wavelength ranges by changing the recording beam wavelength and exposer tilt angle. Furthermore, the demonstrated approach is also amenable to scaling up using laser manufacturing. The holographic arrays can be recorded using different objects (for example, lenses, diffusers, mirrors), and different polymers can be chosen to diffract at different Bragg peaks or act as a holography sensor. We anticipate that wavelength-selective phase-conjugated HCCRs will lead to applications in diffraction grating-based displays, biosensors and components for photonic integrated circuits.

## Author contributions

HB and AKY conceived the idea. RA planned and conducted experiments and simulations, analyzed the results, and wrote the article. HB set up the experiment, supervised the experiments and simulations, and led the project. AKY and SHY made intellectual contributions and AKY edited the article.
