# Supplementary material for: Color-selective holographic retroreflector array for sensing applications
Source: Light Sci Appl. 2017 Feb 24;6(2):e16214–. doi: 10.1038/lsa.2016.214 (PMC6062178; doi:10.1038/lsa.2016.214)
Supplement: Supplementary Information [file lsa2016214x1.docx]

**SUPPLEMENTARY INFORMATION**

Color-selective holographic retroreflector array for sensing applications

Rajib Ahmed,^1^ Ali K. Yetisen,^2,3^ Seok Hyun Yun,^2,3^ and Haider Butt^1^,

^1^Microengineering and Nanotechnology Laboratory, School of Mechanical Engineering, University of Birmingham, Birmingham B15 2TT, United Kingdom

^2^Harvard Medical School and Wellman Center for Photomedicine, Massachusetts General Hospital, 65 Landsdowne St., Cambridge, MA, 02139, USA

^3^Harvard-MIT Division of Health Sciences and Technology, Massachusetts Institute of Technology, Cambridge, MA, 02139, USA

Correspondence: Haider Butt, Email: [h.butt@bham.ac.uk](mailto:h.butt@bham.ac.uk)

**Materials and Equipment.** Glass was used as a substrate and its surface was functionalized with (3-aminopropyl)-triethoxysilane and acetone 1:100 (v/v). To prepare the emulsion, halide salts were formed in DI water by simultaneously adding AgNO_3_ and LiBr in the presence of a photosensitizing dye (quinaldine blue) at constant agitation. The temperature of the solution was increased to 50 °C and AgBr NCs were formed. NC size was controlled with nucleation and concentration of the precursor. The resulting emulsion was coated over the glass substrate using a doctor blade to create ~10 µm film. L-ascorbic acid (99 %), Na_2_CO_3_ (99.9 %) and NaOH (98.0 %) were purchased from Sigma-Aldrich, U.K. 4-methylaminophenol sulfate (metol) (99 %) was purchased from Acros Organics, U.K. The HeNe laser operated at 632.8 nm and about 20 mW of power. The spectrophotometer (resolution of ~0.1-100 nm FWHM) and white light source (450-1100 nm) were purchased from Ocean Optics for reflection measurements. COMSOL Multiphysics (v5.1), and MATLAB (MathWorks, v8.1) were used for finite element simulations and data processing, respectively.

**Holographic Recording of CCRs.** The plates were swollen by immersing them in a triethanolamine bath (10 %, v/v) for a minute. The gelatin surface was wiped with a squeegee to remove triethanolamine from the surface. The plates were left in dark under a flow of cold air for an hour. Room was at ~23 ^o^C and with typical 60 % RH. The HeNe laser was used to produce a collimated beam (Ø = 6 cm), which was reflected down from a front surface mirror, positioned at 45° to illuminate the recording medium which was resting with a slight tilt ( ≤ 5°) on top of the CCR array. The plate was left to settle for 5 min before a 5 s laser light exposure. The plate was subsequently developed and dried. The finished hologram replayed in a bright green color because it had been pre-swollen when exposed.

**Directional, Phase conjugation and Rotation Property of CCR/HCCR Array.**

Suppose an arbitrary incident wave under paraxial approximation (K_z_ = K = $\frac{2\pi}{\lambda}$)

$E_{i}(x,y,z)=A(x,y)\exp(i\varphi(x,y))\exp(-ikz)$ (Eq. S1)

The reflected wave from normal mirror,

$E_{m}(x,y,z)=A(x,y)\exp(i\varphi(x,y))\exp(+ikz)$ (Eq. S2)

In ideal phase conjugation mirror case,

$E_{PC}(x,y,z)=A(x,y)\exp(-i\varphi(x,y))\exp(+ikz)$ (Eq. S3)

In a single corner cube case assuming the size of the corner cube is sufficiently large and the center of the corner cube coincides with the optical axis (z-axis) as shown in Figure S1a, it is given by:

$E_{CC\_Single}(x,y,z)=A(-x,-y)\exp(-i\varphi(-x,-y))\exp(+ikz)a_{CC}(x,y)$ (Eq. S4)

Comparing Eq. (S3) and (S4), we can find that ECC_Single can be approximate as E_PC_ only when amplitude distribution$A(x,y)$is constant inside the aperture of the CC (where $a_{CC}(x,y)$ = 1) and phase distribution $\varphi(x,y)$is that of a plane wave, i.e. $\varphi(x,y)=k_{x}x+k_{y}y$so that -$\varphi(x,y)$ =$\varphi(-x,-y)$.

**Figure S1.** Corner cubes (a) single and (b) array formats

In a corner cube array:

$\begin{matrix} E_{CC\_Array}(x,y,z)= \\ \sum_{m,n} A(2mh-x,2nh-y)\exp(i\varphi(2mh-x,2nh-y))\exp(+ikz)a_{CC}(x,y)rect(\frac{x-mh}{h},\frac{y-nh}{h}) \end{matrix}$(Eq. S5)

where rect(x,y) is a rectangular function having 1 where abs(x,y) < 1/2 and is 0 otherwise.

Comparing Eqs. (S5) and (S3) we can find that ECC_Array can be approximated to ideal phase conjugation E_PC_ if

1. the amplitude variation is slow varying such that it can be considered as constant in each corner cube i.e. $A(x,y)rect(\frac{x-mh}{h},\frac{y-nh}{h})\approx A(mx,ny)rect(\frac{x-mh}{h},\frac{y-nh}{h})$and
2. the phase distribution inside CC area is linear i.e.

$$\varphi(x,y)rect(\frac{x-mh}{h},\frac{y-nh}{h})\approx\varphi(k_{x,m,n}x+k_{y,m,n}y)rect(\frac{x-mh}{h},\frac{y-nh}{h})$$

Eq. (S5) can be written as:

$\begin{matrix} E_{CC\_Array}(x,y,z)= \\ \sum_{m,n} A(mh,nh)\exp(i2mhk_{x,m,n}+i2nhk_{y,m,n}y))\exp(+ikz)a_{CC}(x,y)rect(\frac{x-mh}{h},\frac{y-nh}{h}) \end{matrix}$ (Eq. S6)

where m, n represent each segment (i.e optical field segment just reflected by each corner cube),

$\exp(ik_{x,m,n}x-ik_{y,m,n}y)$ represents the phase conjugation and $\exp(i2mhk_{x,m,n}+i2nhk_{y,m,n})$ is the phase mismatch between the segments.

Figure S2a-d shows arbitrary incident wave, reflected from normal mirror (NM) and single corner cube retroreflector (CCR) and CCR array surface. The amplitude and phase reversal occurred for the single CCR surface (Eq. S4). However array CCR shows reversed phase but same amplitude of incident wave. This is due to the fact that CCR surface consisted of triangular sections (Figure S2e) and 2D view of CCR surface is equivalent to triangular grating structure (side view) (Figure S2f) which helps to split of incident wavefront (Figure S2d). [^1^](#_ENREF_1) Based on diffraction property of the HCCR array we have introduced rotational property of the HCCR to characterize them. If the sample rotates in clockwise direction, the diffracted color light (for broadband light source) from HCCR surface rotates in anticlockwise direction (Figure S2g). If the incident holographic image (for monochromatic light source) rotates in clockwise direction, the far-field projected image from HCCR surface rotates in anticlockwise direction (Figure S2h).

**Figure S2.** Optical properties of Retroreflector. (a) The arbitrary wave-front incident to the normal and CCRs, (b) reflected wave front from the normal mirror, (c) the single CCR and (d) array CCRs surface. (e) CCRs array surface consisted with triangular sections, (f) 2D view of CCRs array surface are equivalent to triangular grating structure (side view). (g-h) Rotational property of the HCCR/CCR: diffracted color light/ projected image rotate in reverse direction of incident light/holographic image.

**Simulation of directional property of CCR array**. The simulation of the directional property of the CCR array was performed with commercial COMSOL muti-physics simulation tool. [^2^](#_ENREF_2) For the simplicity, minimum time and computational memory, 2D simulation was performed with triangular mesh and maximum mesh size 135 nm (one tenth of the incident wavelength). Light propagation was simulated with wavelength (λ = 632 nm) for the reflection mode and high-intensity directional reflection was observed.

Figure S3a shows the geometry of the simulated model for normal light reflection from the triangular grating arrays. Far-field reflection intensity was observed from a hemispherical screen geometry in air (radius *=* 10 µm) surrounding the triangular grating array. By directing 632 nm incident light with three different angles (θ = 0°, +30° and -30°) at the triangular reflector grating, we evaluated reflection intensity spectra for the model retroreflector. Results showed that the incident light was reflected back toward the source independent of the incident angles, which is in agreement with theory. Figure S3b shows the reflected light with respect to angles. With normal incident (*i* = 0°), light reflected strongly toward the source and had less scattering toward different direction. With inclination (*i* = +30° and -30°), both the light reflected from sidewalls and strong directional peaks were observed. The direction of reflections was symmetric. The three peaks at certain incident angle correspond to different diffraction orders. Thus the diffraction angle shows strongly dependent on the incident angles.

The incident (red) and reflected (black) waves from the corner cubes produce an interference pattern, which is dominant near to the CCR’s surface (Figure S3d). Interference occurs in between two triangular sections due to cross directional reflected waves. The interference intensities were plotted across various planes (Figure 3d). Different field intensities were observed between inner (H-Plane 1) and outer (H-Plane 2) regions due to light interference (Figure S3f-g). Analogous results were also found for the vertical plane (V-Plane). Stronger interference was observed nearer to the reflector plates due to side wall reflections.

**Figure. S3.** Computational modeling of CCRs. (a) Simulated geometry. (b) Angular reflection intensity. (c-e) CCRs directional property: light reflected from retroreflector array return toward the source direction and is independent of the incident angles. (f-g) Electric field intensity spectra for the horizontal and vertical planes for a single retroreflector.

**CCR Array and HCCR Array Size.** The holographic recoding of the CCR array was performed with a circular retroreflector. The dimension of each CCR was ~ 0.2 cm. Each CCR consisted of three perpendicular planes and they were split into six planes (Figure S4a-b). The recorded image of the HCCR array was similar to the object CCR array. The dimension of each cell was ~ 0.2 cm. Holographic image strongly depended on viewing angle. Therefore to take better image, the recorded HCCR hologram was tilted during image capture which created a slight difference in cell sizes on the HCCR (lower parts compare to the upper parts) (Figure S4c). Similar six splitted planes also exist in the microscopic image of the recoded HCCR.

**Figure S4.** Corner cube retroreflectors in different formats. (a) Shows bicycle corner cube reflector array and magnified version of a single cell. (b) Splitted CCR planes. (c) HCCR array and its magnified version consisted with splitted planes.

**Temperature and Relative Humidity Sensitivity Measurement.** The spectral response was collected through a spectrophotometer (450-1100 nm, 0.2 nm resolution). Sensitivity of the proposed HCCR sensor can be measured from the response curves. The temperature sensitivity can be measured from the ratio of the change in peak wavelength and temperature variation.

Temperature sensitivity, S_T_ = $\left(\frac{\Delta\lambda}{\Delta T}\right)_{RH=40\%}$ $\approx$ 4 nm / °C (at 23°C)

The relative humidity (RH) means how moist the air in the chamber and define as the ratio of the partial pressure of the water vapor ($p_{H_{2}O}$) to equilibrium pressure of the water vapor ($p_{H_{2}O}^{*}$) at specific temperature and express as percentage, [^3^](#_ENREF_3)

RH (%) = $\frac{P_{H_{2}O}}{P_{H_{2}O}^{*}}$

Similarly, the relative humidity sensitivity can be measured as the ratio between small change of peak wavelength shift and relative humidity variation.

Relative Humidity Sensitivity, S_RH_ = $\left(\frac{\Delta\lambda}{\Delta RH}\right)_{Temp=23^{\circ}C}$ $\approx$1 nm (at 40 % RH)

Sensitivity value will be higher at higher temperature and relative humidity variation. The sensing performance of the HCCR can be performed with a control environment chamber (home made). The relative humidity (RH) and temperature of the chamber can be controlled through a microcontroller system. The microcontroller system is able to precisely control relative humidity and temperature variation of the chamber about 1 % RH and 1°C, respectively.

**Single Color Diffractive HCCR Array and Optical Properties.** Fabrication was based on HeNe laser beam (λ = 632.8 nm, 20 mW) and Denisyuk reflection mode. Experiment setup similar to the HCCR array fabrication in the main article (Figure 1): the object (CCR array) and recoding medium was placed with tilted (θ ≤ 5°) angle. With current experiment setup (Figure S5a), the CCR array and recording medium are parallel. The recorded HCCR also shows district optical characteristics as compared to the sample in the main article.

**Figure S5.** (a) Fabrication of holographic CCR arrays with sample and object are parallel to each other. Scale bar = 2 cm. (b) Normalized monochromatic diffracted light as a function of incident angles; inset shows linear fitting of diffracted peak wavelength as a function of incident angle. (c) Diffracted lights with different receiver position θ, and peak wavelengths ($\lambda_{P}$). Scale bar = 2 cm.

Single color (green) light diffract with white light illumination. The peak wavelength shift left (lower wavelength) with incident variation (Figure S5b) similar to previous (main article). But diffracted color light limited with green wavelength (550-500 nm). Figure S5c shows far-field diffracted light with incident angle variation. In previous (main article), the diffracted color light and reflected white light was separate. But with current experiment, diffracted color light surrounding to the reflected white light and hard to distinct them. Moreover, lower green light exists with larger angle and was hard to measure with spectrometer.

**Figure S6.** (a) Normal reflection (90^o^ illumination) from the CCR surface has been observed with a spectrometer and optical microscope setup. Light incident to corner surface and reflect back to the Ocean Optics 2000 spectrometer (top of the microscope). (b) Reflection property shows that maximum green reflect from the corner surface. (c) Maximum green light intensity for different incident angles. Here, spectrometer position keep fixed (r = 18 degree). (d) Each Diffraction angle (for maximum green light) follow linear relationship with each incident angle.

Further optical experiment was performed with normal white light illumination through objective of an optical microscope setup and spectrometer kept top of the microscope to collect reflected light through same objective lens (Figure S6a). Incident white light (400-1100 nm) illuminate to HCCR surface and reflect back to the spectrometer (top of the microscope). Reflection property shows that maximum green reflect from the HCCR surface (Figure S6b).

Figure S6c shows optical experiment result in which incident angle varies and collection angle kept fix and try to measure maximum diffracted green light. The peak of the diffracted green light shows clockwise rotation with incident angle variation. Further optical experiment was performed with incident angle variation and try to measure angle of the maximum diffracted green light. Result shows the incident light and diffracted angle of the maximum diffracted green light maintain linear relationship each other (Figure S6d).

Optical characterization was performed for the diffracted color light with tilt angle variation. Figure S7 shows colorimetric property of the recorded HCCR array with tilt angle variation ($\theta_{l}$ = 10°, 20°, 30°). The diffracted light shifted toward longer wavelength for the larger tilt angle ($\theta_{l}$ = 30°).

**Figure S7.** Wavelength diffraction with HCCR tilt and incident angles; wavelength shifted toward larger wavelength with higher tilt and angles.

**References**

1. Chipman RA, Shamir J, Caulfield HJ, Zhou QB. Wavefront correcting properties of corner-cube arrays. *Appl Opt* 1988; **27**(15)**:** 3203-3209.

2. Butt H, Yetisen AK, Ahmed R, Yun SH, Dai Q. Carbon nanotube biconvex microcavities. *Appl Phys Lett* 2015; **106**(12)**:** 121108.

3. Lawrence MG. The relationship between relative humidity and the dewpoint temperature in moist air: A simple conversion and applications. *Bulletin of the American Meteorological Society* 2005; **86**(2)**:** 225-233.
